# Students’ Attitudes toward COVID-19 Vaccination: An Inter-University Study from Bulgaria

**DOI:** 10.3390/ijerph19169779

**Published:** 2022-08-09

**Authors:** Martina Moskova, Anelia Zasheva, Metodi Kunchev, Ivan Popivanov, Dimo Dimov, Virsavia Vaseva, Todor Kundurzhiev, Ilia Tsachev, Magdalena Baymakova

**Affiliations:** 1 Faculty of Medicine, Medical University, 9002 Varna, Bulgaria; 2 Faculty of Navigation, Nikola Vaptsarov Naval Academy, 9002 Varna, Bulgaria; 3 Department of Infectious Diseases, Military Medical Academy, 1606 Sofia, Bulgaria; 4 Department of Virology, Military Medical Academy, 1606 Sofia, Bulgaria; 5 Department of Military Medicine, Military Medical Academy, 1606 Sofia, Bulgaria; 6 Department of Disaster Medicine, Military Medical Academy, 1606 Sofia, Bulgaria; 7 Department of Teaching and Science Activity, Military Medical Academy, 1606 Sofia, Bulgaria; 8 Department of Occupational Medicine, Faculty of Public Health, Medical University, 1527 Sofia, Bulgaria; 9 Department of Microbiology, Infectious and Parasitic Diseases, Faculty of Veterinary Medicine, Trakia University, 6000 Stara Zagora, Bulgaria

**Keywords:** Bulgaria, COVID-19, questionnaire-based survey, students, vaccines

## Abstract

In Bulgaria, vaccination coverage against the SARS-CoV-2 virus is low. The reasons for this fact are many and varied. The aim of the present study was to establish what the attitudes towards the COVID-19 vaccination process are among students from various specialties from several Bulgarian universities. In this research, 600 students participated, divided into two groups: Doctor of Medicine (MD) students (n = 300) and non-MD students, i.e., students of specialties, such as mathematics, engineering, finance and economics, law, human sciences, etc. (n = 300). Each respondent completed a questionnaire which was divided into three parts with closed questions. The mean age of all students was 21.19 ± 1.87 years (95% CI: 20.48–21.90). The female sex dominated among the analyzed participants (sex ratio: female/male = 1/0.85). Nearly 62% (371/600) of individuals declared that they have been COVID-19 vaccinated with at least one dose (*p* < 0.001). Overall, 33% of the participants sought information on vaccines from video sharing platforms and 36.0% (216/600) from social media platforms. From the conducted multivariable logistic regression the odds of vaccination against COVID-19 were 6.225 times higher in individuals with a positive attitude towards these vaccines than in people with a negative attitude towards them (*p* < 0.001). We have found that those students who trust the international health organizations had an OR of 2.365 (*p* = 0.004) to be SARS-CoV-2 vaccinated. We estimated that the odds of vaccination against SARS-CoV-2 among children were 4.794 times higher in parents (students) who had been vaccinated than in non-vaccinated parents (students) (*p* < 0.001). Our results could support the national public health organizations, the national educational/scientific systems, and the management of Bulgarian universities in making future decisions about the field of COVID-19 control and prevention.

## 1. Introduction

In early 2020, the first reports of pneumonia of unknown etiology appeared in the Hubei province, China [1,2]. Researchers later found that a new coronavirus was the cause of the growing number of clinical cases of the disease. Initially, the new virus was named 2019-nCoV, and later it was renamed SARS-CoV-2 and the disease it causes was named COVID-19 [3,4]. In mid-March 2020, the World Health Organization (WHO) declared a pandemic of COVID-19. Until 5 July 2022, in the world there were 546,357,444 confirmed cases, 6,336,415 confirmed deaths, and a case-fatality rate (CFR) = 1.15 [4]. Until 5 July 2022, in Bulgaria there were 1,174,216 confirmed cases, 37,260 confirmed deaths, and CFR = 3.17 [5].

By the end of 2020, there were no approved vaccines against COVID-19. The first approved vaccine (BNT162b2 mRNA) was authorized for use by the European Medicines Agency (EMA) on 21 December 2020 and was first delivered in Bulgaria on 26 December 2020 [5,6]. Four more vaccines were later approved in the European Union (EU): mRNA-1273 vaccine (approved by EMA–6 January 2021; delivered to Bulgaria–13 January 2021); ChAdOx1 nCoV-19/AZD1222 vaccine (approved by EMA–29 January 2021; delivered to Bulgaria–7 February 2021); Ad26.COV2-S vaccine (approved by EMA–11 March 2021; delivered to Bulgaria–15 April 2021); and NVX-CoV2373 vaccine (approved by EMA–20 December 2021; not delivered to Bulgaria) [5,6]. The availability of COVID-19 vaccines has led to high vaccination opportunities for the EU population. To date, in the EU/the European Economic Area (EEA) countries have been vaccinated with one dose more than 341 million citizens (75.4%); primary course—329 mln. (72.7%); first booster—238 mln. (52.7%); and second booster—3.2% [7]. For example, the countries in the EU/EEA with the highest COVID-19 vaccination coverage are Portugal (one dose—94.5%; primary course—86.3%; first booster—65.4%; second booster—3.8%), Malta (87.2%; 86.1%; 67.8%; 7.1%), and Spain (87.0%; 78.9%; 53.6%; 0.4%); the countries in the EU/EEA with the lowest vaccination coverage are Bulgaria (30.2%; 29.8%; 11.0%; missing data), Romania (42.6%; 42.3%; 9.1%; 0.0%), and Slovakia (51.7%; 50.8%; 30.3%; 0.1%) [7]. It was established during the pandemic that persons who have been vaccinated less often have a severe clinical form, critical clinical form, and they are less likely to have fatal outcome compared to unvaccinated individuals. This was a very important conclusion for the use of these vaccines because it showed the importance and meaning of their application.

In Bulgaria, vaccination coverage against the SARS-CoV-2 infection is low. The reasons for this fact are many and varied. The aim of the present study was to establish attitudes towards to the vaccination process (towards all vaccines and specifically towards vaccines against the COVID-19) among young and active citizens—students from various specialties from several Bulgarian universities.

## 2. Materials and Methods

### 2.1. Study Design

The present questionnaire designed in Google Forms was sent to 728 random students through email, social media platforms (Facebook, Twitter, and/or Instagram), and the application for messages called Viber. We received a completed questionnaire from 600 students and they were included in the current study. To each participant, it was reported that filling in the questionnaire is voluntary, anonymous, and without compensation (non-remunerated). Informed consent was considered to be obtained through the act of responding. Only fully completed questionnaires were included in our analysis. The questionnaire was validated by the infectious disease specialist (physician) and clinical virology specialist (physician).

### 2.2. Sample Size

**Figure 1:**
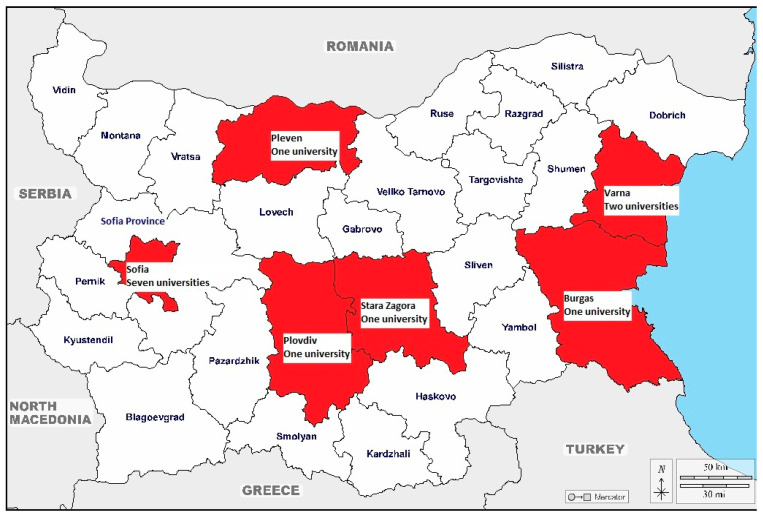
Geographic location of the Bulgarian universities from which there were respondents in the present study (in red).

According to the database of Bulgarian Ministry of Education and Science there are 52 universities in the country (Register of Higher Education Institutions in the Republic of Bulgaria) [8]. In this research, 600 students participated (n = 600), divided into two groups (randomly selected): Doctor of Medicine (MD) students (n = 300) and non-MD students, i.e., students of specialties, such as mathematics, engineering, finance and economics, law, human sciences, etc. (n = 300). The group of MD students included participants from the medical faculties of Medical University of Pleven, Medical University of Plovdiv, Medical University of Sofia, Medical University of Varna, St. Kliment Ohridski Sofia University, and Trakia University of Stara Zagora (Figure 1). The group of non-MD students included participants from the non-medical faculties of Krastyo Sarafov National Academy for Theater and Film Arts of Sofia, Ministry of Interior Academy of Sofia, Nikola Vaptsarov Naval Academy of Varna, Prof. Assen Zlatarov University of Burgas, St. Kliment Ohridski Sofia University, Technical University of Sofia, University of National and World Economy of Sofia, and Vassil Levski National Sports Academy of Sofia. The study was conducted in the period from 1 February 2022 to 30 March 2022.

### 2.3. The Questionnaire

The current questionnaire was divided into three parts with closed questions. The first section consisted of questions related to the participant, including their age, sex, place of residence, ethnicity, religion, marital status, tobacco smoking, and frequency of alcohol consumption. Regarding the place of residency we divided the cities in 3 groups: a “small city” with a population up to 25,000 citizens; a “middle city” of between 25,000 and 100,000 citizens; and a “large city” of more than 100,000 citizens. The term “unspecified ethnicity” in the manuscript refers to participants who were part of a small ethnic group (Gypsy, Jewish, Armenian, and others). The phrase “unspecified religion” in the present study refers to students who were part of small religious communities in the country (Judaism, Catholicism, etc.). The term “tobacco smoking” in this survey refers to participants who have used various tobacco products (classic cigarettes, smokeless cigarettes, pipe smoking, etc.). The phrase “alcohol consumption” in the manuscript refers to participants who have used products with a high alcohol content (whiskey, rakia/fruit brandy, vodka, ouzo, etc.) and does not include the consumption of beer and/or wine. The second section consisted of questions addressing the students’ attitudes towards all vaccine-related matters, such as application of all mandatory vaccines; obtaining information on vaccines (radio, television, social media platforms, video sharing platforms, etc.); and knowledge of the scientific journals in their field. The third section involved questions about students’ attitudes toward COVID-19 vaccines specifically, such as their opinion on these vaccines; sufficient scientific and laboratory testing of COVID-19 vaccines; hiding information from manufacturers and national health authorities about the risks of these vaccines; hiding information from international health organizations (WHO, European Centre for Disease Prevention and Control—ECDC, etc.) about the risks of these vaccines; trust in the information on COVID-19 vaccines provided by national health organizations and international health organizations (WHO, ECDC, etc.); whether they had been vaccinated against COVID-19; and whether they would vaccinate their child against COVID-19.

### 2.4. Statistical Analysis

All basic and demographic characteristics derived from our questionnaire were compared by T-test and the Chi-square test. The results from the second and third part of the questionnaire were compared by the Chi-square test. The Z-test was used to test the hypothesis that the observed proportion was equal to a predetermined proportion. Univariate logistic regression was used to evaluate the risk between the COVID-19 vaccinated students and various indicators. Multivariable logistic regression (forward conditional) was applied to assess the relationship between students vaccinated against SARS-CoV-2 infection and some parameters. Statistical analysis was performed by SPSS Statistics 20.0 (IBM Corp., Armonk, NY, USA) and Excel 2007 (Microsoft, Redmond, WA, USA). A *p*-value < 0.05 was considered statistically significant.

### 2.5. Ethical Considerations

The current questionnaire-based research was designed to assess Bulgarian students’ attitudes toward COVID-19 vaccination. It was conducted by a multidisciplinary scientific team of Military Medical Academy, Sofia, Bulgaria, and researchers by other scientific organizations from Bulgaria. The survey was approved by the Local Ethics Committee of Military Medical Academy, 1606 Sofia, Bulgaria (1 January 2022), who confirmed that the research was in full accordance with ethical principles included in the World Medical Association Declaration of Helsinki (adopted in June 1964, last revision in October 2013). The present research was performed following the Strengthening the Reporting of Observational Studies in Epidemiology (STROBE) guidelines [9].

## 3. Results

In the present study, were included 600 students with mean age 21.19 ± 1.87 years (95% CI: 20.48–21.90); male–21.05 ± 1.78 years (95% CI: 20.05–22.05); and female–21.31 ± 1.93 years (95% CI: 20.32–22.30). The female sex dominated among analyzed participants (sex ratio: female/male = 1/0.85). Persons living in rural areas (village) represented 5.5%, those living in urban areas (city) represented 94.5%, while 62.7% lived in large towns (Table 1). The main part of the participants declared that they were of Bulgarian ethnic origin (92.7%; 556/600), and a small part were of Turkish ethnic origin (5.2%; 31/600) or unspecified ethnicity (2.1%; 13/600). In addition, the majority of students profess the Eastern Orthodox religion (70.7%), followed by the non-religious persons (14.0%), unspecified religion (9.2%), and Islam (6.1%). Regarding marital status, it was observed that the predominant part of the participants was single or had a boyfriend/girlfriend. Non-smokers dominated among analyzed students (smoking ratio: non-smoking/smoking = 1/0.48; *p* < 0.001). Persons who consumed alcohol were almost twice that of those who did not consume (64.5% vs. 35.5%; *p* < 0.001).

Overall, 71% (428/600) of the participants had a positive attitude towards vaccines and the vaccination process (Table 2). The number of students who received all mandatory vaccines (part of the National Vaccination Calendar in Bulgaria) was about five times greater than those with omissions in mandatory vaccination status (82.8% vs. 17.2%; *p* < 0.001). Approximately half of the persons sought information about vaccines on radio and television broadcasts (52.7%; 316/600). Overall, 33% (198/600) of the participants sought information on vaccines from video sharing platforms (YouTube, Vbox7, etc.) and 36.0% (216/600) from social media platforms (Facebook, Twitter, Instagram, etc.). Just under half of the students looked for information in news websites (46.2%; 277/600). Overall, 55% (334/600) of individuals reported seeking information about vaccines from specialized literature (peer-reviewed journals, textbooks); 66.0% from a general practitioner (GP) or physician specialist (396/600; *p* = 0.006); and 72.3% from friends and family (434/600; *p* = 0.045). Almost two-thirds of the participants (61.0%; 366/600) did not know about peer-reviewed journals and only 2.5% (15/600) knew about more than 10 peer-reviewed journals in their professional field (*p* < 0.001).

Approximately half of the students had positive attitude towards the COVID-19 vaccines (positive vs. negative = 54.8% vs. 45.2% = 329/600 vs. 271/600) (Table 3). Overall, 67% (403/600) of participants thought that these vaccines had not been tested enough (scientific and laboratory). An equal percentage (62.3%; 374/600; *p* < 0.001) of individuals thought that SARS-CoV-2 vaccine manufacturers and national governments (with their health organizations) hide information about the risks of the application these vaccines. Half of the students (50.3%; 302/600) did not trust the information about COVID-19 vaccines received from international health organizations (WHO, ECDC, etc.). In addition, most participants did not trust the information on SARS-CoV-2 vaccines received from national health organizations (distrust vs. trust = 54.5% vs. 45.5% = 327/600 vs. 273/600). Almost 62% (371/600) of individuals declared that they had been COVID-19 vaccinated with at least one dose (*p* < 0.001), without saying whether they had primary course and/or first booster/second booster. Furthermore, a high percentage of participants said that they will not vaccinate their children against SARS-CoV-2 infection (62.7%; 376/600; *p* < 0.001).

From the conducted univariate logistic regression, a positive correlation between COVID-19 vaccination and the use of information from peer-reviewed journals/textbooks was found (*p* = 0.002; OR = 1.697) (Table 4). Students using information from a GP or physician specialist had 1.460 times the odds of SARS-CoV-2 vaccination compared with those who were not informed by these sources (*p* = 0.032; 95% CI: 1.034–2.061). Individuals with a positive attitude towards these vaccines had 22.262 times the odds of vaccination against COVID-19 compared with those with a negative attitude towards (*p* < 0.001). We had found that a very large part of those who trust the national health organizations (*p* < 0.001; OR = 12.593) and the international health organizations (*p* < 0.001; OR = 14.848) had been SARS-CoV-2 vaccinated. Parents (students) who had been vaccinated had 24.111 times the odds of vaccination against SARS-CoV-2 among their children compared with non-vaccinated parents (students) (*p* < 0.001).

Multivariable logistic regression estimated the relationship between vaccinated COVID-19 students and some indicators (Table 5). Predictor factors for COVID-19 vaccination were the positive attitude towards SARS-CoV-2 vaccines, the trust of international health organizations, being residents of cities, and positive attitude towards vaccinating children against SARS-CoV-2. The residents of cities had 3.322 times the odds of COVID-19 vaccination, and those with positive attitude towards vaccinating children had an OR of 4.794 (*p* < 0.001). Students with a positive attitude towards COVID-19 vaccine had 6.225 times the odds of COVID-19 vaccination, and those who trust international health organizations had an OR of 2.365 (*p* = 0.004).

## 4. Discussion

The COVID-19 pandemic surprised governments and national health systems around the world. Many of them were not prepared for this serious medical challenge. Bulgaria and the Bulgarian public health system were also in a difficult situation. In this regard, several studies on SARS-CoV-2 infection had been conducted in our country, including: a seroepidemiological survey [10]; research on clinical characteristics of patients with COVID-19 [11,12]; studies of immune response to SARS-CoV-2 vaccines [13,14,15]; research into adverse events of COVID-19 vaccines [16]; studies dealing with inconclusive SARS-CoV-2 PCR samples [17]; and studies of liver involvement in children with COVID-19 and multisystem inflammatory syndrome [18]. The Bulgarian vaccination process started on 26 December 2020. A national vaccination plan was prepared, including five phases of vaccination, which were to cover the entire population of the country. Despite the efforts of the Bulgarian national health authorities and the national vaccination committee, vaccination coverage in Bulgaria is still very low (one dose—30.2%; primary course—29.8%; first booster—11.0%; second booster—missing data). In this regard, we conducted the present study to try to identify some of the reasons for this fact among the young and active population, namely students from several Bulgarian universities.

In the countries of south-eastern Europe, several scientific teams had worked on the topic of attitude towards the vaccines and the vaccination process. A study from Greece reported that 89.3% of participants who trusted the information received from the Greek Public Health authorities were vaccinated against SARS-CoV-2 infection (*p* < 0.001) [19]. The same authors found that physicians who received information about the SARS-CoV-2 vaccines by social media platforms registered lower rates of vaccination coverage against COVID-19 [19]. In the Romanian survey of Balan et al., it was reported that over 88% of all students had a positive attitude towards COVID-19 vaccination [20]. The main reason for SARS-CoV-2 vaccine rejection declared by Romanian healthcare students was related to the faster development of COVID-19 vaccines (*p* < 0.001). A large part of the participants take information about these vaccines from official sources: the Romanian national vaccination campaign (70.9%), peer-reviewed journals (69.3%), lectures of university teachers (62.8%), the Romanian Ministry of Health web page (53.5%); social media platforms were the least popular source [20]. Furthermore, about 89.0% of these Romanian respondents trust the safety and efficacy of SARS-CoV-2 vaccines. Research from Croatia by Bagic et al. reports that the main reasons for vaccine hesitancy were the fact that people think that the SARS-CoV-2 vaccines were not sufficiently safe (82%; 627/765), and distrust in vaccines in general (71%; 543/765) [21]. In addition, these Croatian scientists had established a relationship between trust in researchers and hesitation about vaccines: higher trust in scientists means less hesitation in vaccines. In the same research, the authors found that distrust in the media led to a 2-times increase in the likelihood of hesitancy for COVID-19 vaccines [21]. In addition, those who distrust the national government were 1.5 times more likely to be hesitant. In a survey from North Macedonia, it was found that, in all research models (from model 1 to model 3), age remained significantly associated with SARS-CoV-2 vaccine hesitancy; increased age was associated with decreased odds of COVID-19 vaccine hesitancy (OR-value decreases with increasing age) [22]. The same authors found that misinformation about SARS-CoV-2 vaccines did not have a statistically significant effect on those who were hesitant and those who were non-hesitant [22]. Turkish authors reported that younger participants preferred the internet and multiple sources as references for information about COVID-19 vaccines compared to older individuals who preferred information from television (*p*  <  0.001) [23]. The same research team found that younger persons were worried that the SARS-CoV-2 vaccines had not been administered to sufficient citizens before, while the older individuals were more worried about the COVID-19 vaccine’s side effects (*p*  =  0.044) [23].

In a global cross-sectional study by Riad et al., among over 6000 students from 22 countries who participated, including from the following Balkan countries: Albania—0.9% (57/6639), Croatia—2.5% (169/6639), and Turkey—5.8% (386/6639) [24]. The research includes 22 countries with different economic development (according to the World Bank Atlas method): low- and lower-middle-income economies (LLMI) and upper-middle- and high-income economies (UMHI). The LLMI students were more hesitant to take the SARS-CoV-2 vaccine in comparison to their peers in UMHI countries (30.4% vs. 19.8%, respectively; *p* < 0.01) [24]. In addition, LLMI participants were more significantly influenced by news in media and social media platforms than their UMHI peers (42.0% vs. 30.4%, respectively; *p* < 0.01). Furthermore, UMHI individuals had significantly more confidence in government and pharmaceutical companies (37.9% and 51.0%, respectively; *p* < 0.01) than LLMI persons (27.1% and 37.0%, respectively; *p* < 0.01) [24]. The UMHI students were significantly more confident that they had enough information about SARS-CoV-2 vaccines than LLMI students (33.1% vs. 27.0%, respectively; *p* < 0.01). In this regard, the best levels of acceptance of COVID-19 vaccines were in high-income countries (4.36 ± 1.07), and the lowest levels of acceptance were in low-income countries (3.37 ± 1.17) [24]. Riad et al. also found that higher dependence on media and social media platforms was significantly associated with lower SARS-CoV-2 vaccine acceptance (*p* < 0.01); higher confidence in the government and the pharmaceutical companies was significantly associated with a higher level of COVID-19 vaccine acceptance (*p* < 0.01); lower levels of SARS-CoV-2 vaccines acceptance were significantly associated with lower knowledge of SARS-CoV-2 vaccines safety (*p* < 0.01) [24].

Results from the present study were identical to those in other Balkan countries. Students receiving information about vaccines from the specialized literature (peer-reviewed journals and textbooks) and from a GP (or physician specialist) were more likely to be vaccinated against SARS-CoV-2 infection 1.697 times (*p* = 0.002; 95% CI: 1.217–2.366) and 1.460 times (*p* = 0.032; 95% CI: 1.034–2.061), respectively, in comparison to the uninformed persons. In addition, we found that there is a significant impact between knowledge of 2–3 peer-reviewed journals with IF in the professional field and COVID-19 vaccination rates (OR = 1.735; *p* = 0.004). Therefore, we can conclude that, potentially, students who search and receive information from reliable sources were much more likely to be vaccinated against SARS-CoV-2 infection in comparison to students who do not use reliable sources. In this regard, Serbezova et al. report that 38.0% of the 480 analyzed students had been vaccinated against COVID-19 [25]. The type of vaccine was important for 23.0% of vaccinated individuals and for 40.0% of unvaccinated persons [25]. In the same survey, participants reported they were informed about SARS-CoV-2 vaccines from the internet (30.2%; 145/480), a medical specialist (22.1%; 106/480), a GP (13.1%; 63/480), friend/family (8.1%; 39/480), television/radio (6.9%; 33/480), etc. [25]. Kineva reported that only 10.0% of individuals were informed about SARS-CoV-2 vaccination by peer-reviewed articles and 7.0% by GP and physician specialist [26]. In the same research, it was reported that a large part of participants received information about COVID-19 vaccines from the media (30.0%) and social media platforms (28.0%) [26].

In our survey, the fact that 70.3% of non-MD students and 51.7% of MD students do not know about peer-reviewed journals with IF in their professional field (*p* < 0.001) inspires serious concern. This is a serious problem because the science and the research activity go “*hand in hand*” with the quality of education in universities and scientific organizations. That is why the Bulgarian national education/science authorities and the management of Bulgarian universities must make serious efforts in this direction.

Another interesting result of our study was the fact that 61.8% of students were vaccinated against SARS-CoV-2 (MD students—73.3%; non-MD students—50.3%). This result differs from the COVID-19 vaccination coverage in our country, which was 30.2%. The reasons for this may be different. First, we do not know what part of the participants in the research had one dose, primary course, and/or the first booster/second booster. Such information was not sought by the students so that we could maximize their confidentiality and be able to persuade more of them to participate in our study. Second, from the beginning of the academic 2021–2022 year, to visit most of the Bulgarian universities required the “EU Digital COVID Certificate” (especially for the medical faculties). Most likely, this has increased SARS-CoV-2 vaccination coverage among students. Third, students were probably better informed about the benefits of COVID-19 vaccines in comparison to the general population. This cannot be established precisely because there was no detailed and in-depth peer-reviewed survey of the Bulgarian general population attitudes toward the COVID-19 vaccination. Fourth, probably part of the respondents had given an answer that they had been vaccinated without this being true, due to public pressure from various institutions for vaccination against SARS-CoV-2.

Trust is a key factor, both in human relationships and in various international organizations, national structures, state bodies, public systems, and non-governmental organizations. The data from the current research (univariate logistic regression) showed that students who had trust in national health organizations and international health organizations were 12.593 times (*p* < 0.001; 95% CI: 8.114–19.544) and 14.848 times (*p* < 0.001; 95% CI: 9.638–22.875), respectively, more likely to be SARS-CoV-2 vaccinated in comparison to students without trust. The following multivariable logistic regression estimated that students who had trust in international health organizations had 2.365 times the odds of COVID-19 vaccination (*p* = 0.004; 95% CI: 1.307–4.279). The topic of trust is directly related to the topic of hiding information from various organizations. This corresponds to beliefs in conspiracies and conspiracy theories that are more common in low-income economies and lower-middle-income economies due to the lower level of education and science in these countries [27]. In this regard, the results of our study showed that participants who do not think that manufacturers and governments (and their national health organizations) hide information about COVID-19 vaccines were 8.656 times (*p* < 0.001) and 6.152 times (*p* < 0.001), respectively, more likely to be vaccinated against SARS-CoV-2 infection than their peers who think there was hidden information.

The present research has some limitations that need to be addressed. First, the study included participants from several universities, i.e., this was not nationwide survey. Second, we had information on how many of the students had been vaccinated against SARS-CoV-2 infection, but we do not know how many of them had been vaccinated with one dose, primary course, and/or first booster/second booster. Third, the survey covers attitudes in a certain “time window” (the survey period), i.e., attitudes are a dynamic quantity that changes over time. Fourth, the study includes respondents in a narrow age range (because they were students), so we had no information on attitudes among other age groups in the Bulgarian population. Despite these limitations, this research has its merits. This is the first Bulgarian study that includes a comparison between MD students and students from other university specialties (mathematics, engineering, finance and economics, law, etc.) about their attitudes toward COVID-19 vaccination. In addition, our survey includes detailed information on demographic characteristics (sex, age, ethnicity, religion, etc.), attitudes towards mandatory vaccines, attitudes towards SARS-CoV-2 vaccines, trust in different organizations, information from different sources, etc.

## 5. Conclusions

The present study showed Bulgarian students’ attitudes toward SARS-CoV-2 vaccination. We found a high rate of COVID-19 vaccination among respondents (especially among MD students) and a low level of knowledge for peer-reviewed journals with IF in their professional field. Furthermore, the trust in international and national health organizations was corresponding to the level of vaccination: higher trust = more vaccinations and vice versa. Furthermore, for the first time on this topic (attitudes toward COVID-19 vaccination) students from two different groups were compared: MD students and students from other specialties (engineering, law, mathematics, finance and economics, etc.). The data from the study complement the knowledge about the process of vaccination against SARS-CoV-2 in Bulgaria. Our results could support the national public health organizations, the national educational/scientific systems, and the management of Bulgarian universities about making future decisions in the field of COVID-19 control and prevention.

## Figures and Tables

**Table 1:** Basic characteristics among Bulgarian MD students and Bulgarian non-MD students.

Basic Characteristics	MD Students (n = 300)	Non-MD Students (n = 300)	Statistics *	df	*p*-Value
**Age, Years, Mean ± SD**	21.5 ± 2.0	20.8 ± 1.6	4.18	598	<0.001
**Sex, n (%)**					
Male	112 (37.3)	165 (55.0)	18.84	1	<0.001
Female	188 (62.7)	135 (45.0)			
**Place of residence, n (%)**					
Village	20 (6.6)	13 (4.3)	8.20	3	0.042
City, small	29 (9.6)	39 (13.0)			
City, middle	84 (28.0)	59 (19.6)			
City, large	167 (55.8)	189 (63.1)			
**Ethnicity, n (%)**					
Bulgarian	265 (88.4)	291 (97.0)	22.23	2	<0.001
Turkish	25 (8.3)	6 (2.0)			
Unspecified	10 (3.3)	3 (1.0)			
**Religion, n (%)**					
Eastern Orthodoxy	200 (66.8)	224 (74.8)	16.8	3	<0.001
Atheism	44 (14.6)	40 (13.3)			
Unspecified religion	27 (9.0)	28 (9.3)			
Islam	29 (9.6)	8 (2.6)			
**Marital status, n (%)**					
Married	8 (2.6)	6 (2.0)	1.29	3	0.732
Family life without marriage	17 (5.6)	14 (4.6)			
Boyfriend/girlfriend	127 (42.3)	139 (46.3)			
Single	148 (49.5)	141 (47.1)			
**Tobacco smoking, n (%)**					
Yes	72 (24.0)	124 (41.3)	20.49	1	<0.001
No	228 (76.0)	176 (58.7)			
**Frequency of alcohol consumption, n (%)**					
2–3 times per weeks	12 (4.0)	34 (11.3)	22.73	2	<0.001
2–3 times per months	158 (52.7)	183 (61.0)			
Never	130 (43.3)	83 (27.7)			

**Note:** MD—Doctor of Medicine; df—degrees of freedom; SD—standard deviation. * Statistics = Chi-square test or *t*-test.

**Table 2:** Comparison of students’ questionnaire answers for vaccination process and all mandatory vaccines.

Variables	MD Students (n = 300)	Non-MD Students (n = 300)	Statistics *	df	*p*-Value
**What is your attitude to the vaccines? n (%)**					
Positive	252 (84.0)	176 (58.7)	47.08	1	<0.001
Negative	48 (16.0)	124 (41.3)			
**Have you received all mandatory vaccines (according to the Bulgarian National Vaccination Calendar)? n (%)**					
Yes	273 (91.0)	224 (74.7)	28.14	1	<0.001
No	27 (9.0)	76 (25.3)			
**Are you informed for the vaccines from radio and television? n (%)**					
Yes	162 (54.0)	154 (51.3)	0.43	1	0.513
No	138 (46.0)	146 (48.7)			
**Are you informed for the vaccines from online video sharing platforms (YouTube, Vbox7, etc.)? n (%)**					
Yes	106 (35.3)	92 (30.7)	1.48	1	0.224
No	194 (64.7)	208 (69.3)			
**Are you informed for the vaccines from online social media platforms (Facebook, Twitter, Instagram, etc.)? n (%)**					
Yes	107 (35.7)	109 (36.3)	0.03	1	0.865
No	193 (64.3)	191 (63.7)			
**Are you informed for the vaccines from news websites? n (%)**					
Yes	140 (46.7)	137 (45.7)	0.06	1	0.806
No	160 (53.3)	163 (54.3)			
**Are you informed for the vaccines from specialized literature (peer-reviewed journals, textbooks on the topic)? n (%)**					
Yes	217 (72.3)	117 (39.0)	67.53	1	<0.001
No	83 (27.7)	183 (61.0)			
**Are you informed for the vaccines by talking with friends and family? n (%)**					
Yes	206 (68.7)	228 (76.0)	4.03	1	0.045
No	94 (31.3)	72 (24.0)			
**Are you informed for the vaccines by talking with general practitioner (GP) or physician specialist? n (%)**					
Yes	214 (71.3)	182 (60.7)	7.61	1	0.006
No	86 (28.7)	118 (39.3)			
**How many peer-reviewed journals with Impact Factor (IF) can you list in your professional field? n (%)**					
>10 journals	7 (2.3)	8 (2.7)	27.15	3	<0.001
7–8 journals	18 (6.0)	19 (6.3)			
2–3 journals	120 (40.0)	62 (20.7)			
I don’t know	155 (51.7)	211 (70.3)			

**Note:** * Statistics = Chi-square test.

**Table 3:** Comparison of students’ questionnaire answers for COVID-19 vaccination process and SARS-CoV-2 vaccines.

Variables	MD Students (n = 300)	Non-MD Students (n = 300)	Statistics *	df	*p*-Value
**What is your attitude to the COVID-19 vaccines? n (%)**					
Positive	203 (67.7)	126 (42.0)	39.90	1	<0.001
Negative	97 (32.3)	174 (58.0)			
**Have you been vaccinated against COVID-19? n (%)**					
Yes	220 (73.3)	151 (50.3)	33.62	1	<0.001
No	80 (26.7)	149 (49.7)			
**Do you think that the COVID-19 vaccines had been enough tested (scientific and laboratory)? n (%)**					
Yes	129 (43.0)	68 (22.7)	28.12	1	<0.001
No	171 (57.0)	232 (77.3)			
**According to you, do the manufacturers of COVID-19 vaccines hide information about the risks of vaccines’ administration? n (%)**					
Yes	147 (49.0)	227 (75.7)	45.43	1	<0.001
No	153 (51.0)	73 (24.3)			
**According to you, do the different governments and national health organizations hide information about the risks of vaccines’ application? n (%)**					
Yes	155 (51.7)	219 (73.0)	29.08	1	<0.001
No	145 (48.3)	81 (27.0)			
**Do you trust the COVID-19 vaccines information shared by international health organizations (WHO, ECDC, etc.)? n (%)**					
Yes	189 (63.0)	109 (36.3)	42.67	1	<0.001
No	111 (37.0)	191 (63.7)			
**Do you trust the COVID-19 vaccines information shared by national health organizations? n (%)**					
Yes	178 (59.3)	95 (31.7)	46.30	1	<0.001
No	122 (40.7)	205 (68.3)			
**Would you vaccinate your child against COVID-19? n (%)**					
Yes	152 (50.7)	72 (24.0)	45.59	1	<0.001
No	148 (49.3)	228 (76.0)			

**Note:** * Statistics = Chi-square test.

**Table 4:** Univariate logistic regression showing the relationship between students vaccinated against COVID-19 and various parameters.

Parameter	n	COVID-19 Vaccinated, n (%)	PE	SE	*p*-Value	OR	95% CI
**Sex**							
Male	277	164 (59.2)				1.000	
Female	323	207 (64.1)	0.207	0.169	0.220	1.230	0.884–1.711
**Place of residence**							
Village	33	16 (48.5)				1.000	
City	567	356 (62.8)	0.644	0.365	0.078	1.903	0.931–3.890
**Ethnic group**							
Bulgarian	556	340 (61.2)				1.000	
Turkish	31	23 (74.2)	−0.602	0.420	0.151	1.826	0.803–4.157
**Marital status**							
Married and Family life without marriage	45	23 (51.1)				1.000	
Boyfriend/girlfriend	266	159 (59.8)	0.352	0.323	0.277	1.421	0.754–2.679
Single	289	189 (65.4)	0.592	0.323	0.067	1.808	0.960–3.404
**Smoking**							
Yes	196	120 (61.2)				1.000	
No	404	251 (62.1)	0.038	0.179	0.831	1.039	0.732–1.475
**Alcohol consumption**							
Yes	387	247 (63.8)				1.000	
No	213	124 (58.2)	−0.236	0.175	0.176	0.790	0.561–1.112
**Information about COVID-19 vaccines from radio and TV**							
No	284	183 (64.4)				1.000	
Yes	316	188 (59.5)	−0.210	0.169	0.214	0.811	0.582–1.129
**Information about COVID-19 vaccines from YouTube, Vbox7, etc.**							
No	402	253 (62.9)				1.000	
Yes	198	118 (59.6)	−0.141	0.178	0.429	0.869	0.613–1.231
**Information about COVID-19 vaccines from Facebook, Twitter, Instagram, etc.**							
No	384	247 (64.3)				1.000	
Yes	216	124 (57.4)	−0.291	0.174	0.095	0.748	0.532–1.051
**Information about COVID-19 vaccines from news websites**							
No	323	200 (61.9)				1.000	
Yes	277	171 (61.7)	−0.008	0.169	0.963	0.992	0.713–1.381
**Information about COVID-19 vaccines from specialized literature (peer-reviewed journals, textbooks on the topic)**							
No	266	146 (54.9)				1.000	
Yes	334	225 (67.4)	0.529	0.170	0.002	1.697	1.217–2.366
**Information about COVID-19 vaccines obtained by talking with friends and family**							
No	166	110 (66.3)				1.000	
Yes	434	261 (60.1)	−0.264	0.191	0.168	0.768	0.528–1.117
**Information about COVID-19 vaccines obtained by talking with GP or physician specialist**							
No	204	114 (55.9)				1.000	
Yes	396	257 (64.9)	0.378	0.176	0.032	1.460	1.034–2.061
**Students know the peer-reviewed journals with IF in their professional field**							
I don’t know	366	209 (57.1)				1.000	
2–3 journals	182	127 (69.8)	0.551	0.193	0.004	1.735	1.188–2.532
7–8 journals	37	26 (70.3)	0.574	0.375	0.126	1.776	0.852–3.702
>10 journals	15	9 (60.0)	0.119	0.538	0.824	1.127	0.393–3.231
**Attitude towards COVID-19 vaccines**							
Negative	271	76 (28.0)				1.000	
Positive	329	295 (89.7)	3.103	0.226	<0.001	22.262	14.295–34.670
**COVID-19 vaccines have been laboratory tested enough and sufficient scientific proof exists**							
No	403	191 (47.4)				1.000	
Yes	197	180 (91.4)	2.464	0.273	<0.001	11.752	6.887–20.054
**Manufacturers of COVID-19 vaccines hide information about the risks of vaccines’ administration**							
Yes	374	172 (46.0)				1.000	
No	226	199 (88.1)	2.158	0.230	<0.001	8.656	5.517–13.582
**Different governments and national health organizations hide information about the risks of vaccines’ application**							
Yes	374	179 (47.9)				1.000	
No	226	192 (85.0)	1.817	0.213	<0.001	6.152	4.053–9.338
**Trust the COVID-19 vaccines information shared by international health organizations (WHO, ECDC, etc.)**							
No	302	106 (35.1)				1.000	
Yes	298	265 (88.9)	2.698	0.220	<0.001	14.848	9.638–22.875
**Trust the COVID-19 vaccines information shared by national health organizations**							
No	327	128 (39.1)				1.000	
Yes	273	243 (89.0)	2.533	0.224	<0.001	12.593	8.114–19.544
**Would you vaccinate your child?**							
No	376	159 (42.3)				1.000	
Yes	224	212 (94.6)	3.183	0.315	<0.001	24.111	13.016–44.665

**Note:** PE—parameter estimate; SE—standard error; OR—odds ratio; CI—confidence interval.

**Table 5:** Multivariable logistic regression showing the relationship between students vaccinated against SARS-CoV-2 infection and some parameters.

Parameter	n	SARS-CoV-2 Vaccinated, n (%)	PE	SE	*p*-Value	OR	95% CI
**Place of residence**							
Village	33	16 (48.5)				1.000	
City	567	356 (62.8)	1.201	0.538	0.026	3.322	1.159–9.527
**Attitude towards COVID-19 vaccines**							
Negative	271	76 (28.0)				1.000	
Positive	329	295 (89.7)	1.829	0.302	<0.001	6.225	3.446–11.244
**Trust the COVID-19 vaccines information shared by international health organizations (WHO, ECDC, etc.)**							
No	302	106 (35.1)				1.000	
Yes	298	265 (88.9)	0.861	0.303	0.004	2.365	1.307–4.279
**Would you vaccinate your child?**							
No	376	159 (42.3)				1.000	
Yes	224	212 (94.6)	1.567	0.377	<0.001	4.794	2.288–10.044

## Data Availability

Not applicable.
